# Potential Benefit of Retrospective Use of Neutron Monitors in Improving Ionising Radiation Exposure Assessment on International Flights: Issues Raised By Neutron Passive Dosimeter Measurements and EPCARD Simulations During Sudden Changes in Solar Activity

**DOI:** 10.2478/aiht-2020-71-3403

**Published:** 2020-06-29

**Authors:** Marina Poje Sovilj, Branko Vuković, Vanja Radolić, Igor Miklavčić, Denis Stanić

**Affiliations:** 1 Josip Juraj Strossmayer University of Osijek, Department of Physics, Osijek, Croatia

**Keywords:** aviation route doses, cosmic radiation, EPCARD, nuclear track-etched detector, solar activity, detektor nuklearnih tragova, doze na zrakoplovnim letovima, EPCARD, kozmičko zračenje, solarna aktivnost

## Abstract

Since air transport became more accessible, more and more people have been exposed to ionising radiation of cosmic origin. Measuring the neutron dose equivalent is a good approximation of total ambient dose equivalent, as neutrons carry about 50 % of the dose at flight altitudes. The aim of our study was to compare our measurements of the neutron component of secondary cosmic radiation dose, taken with passive dosimeters, with the data obtained from a simulation generated by EPCARD software, which is common in assessing flight crew exposure to ionising radiation. We observed deviations (both above and below) from the expected proportion of the neutron component (between 40 and 80 %), which pointed to certain issues with actual passive dosimeter measurement and the EPCARD simulation. The main limitation of the dosimeter are large uncertainties in high energy neutron response, which may result in underestimation of neutron dose equivalent. The main drawback of the software simulation is monthly averaging of solar potential in calculations, which can neglect sporadic high energy events. Since airlines worldwide almost exclusively use software (due to costs and convenience) to estimate the dose received by their crew, it is advisable to retrospectively recalculate the dose taking into account neutron monitor readings when solar activity changes.

## Abstract (hr)

Budući da je zračni promet postao dostupniji, sve je više ljudi izloženo ionizirajućem zračenju kozmičkoga podrijetla. Neutronska komponenta sekundarnoga kozmičkog zračenja mjerena je pasivnim dozimetrom koji se sastoji od čvrstog detektora nuklearnih tragova i konvertera. Dobiveni rezultati uspoređeni su s podatcima dobivenima simulacijskim softverom EPCARD. Mjerenje neutronskoga doznog ekvivalenta na zrakoplovnim visinama dobra je aproksimacija ukupnog ambijentalnog doznog ekvivalenta, jer na zrakoplovnim visinama neutroni pridonose s oko 50 % doze. Međutim, uočena su određena odstupanja (i iznad i ispod) ove vrijednosti zbog varijacija u Sunčevoj aktivnosti. Ta su odstupanja očita kada se usporede izmjereni neutronski dozni ekvivalent i ukupni ambijentalni dozni ekvivalent dobiven simulacijom. Glavno ograničenje dozimetra velika je nesigurnost u području visokoenergetskih neutrona, što može rezultirati podcjenjivanjem neutronskoga doznog ekvivalenta. Glavni je nedostatak softverske simulacije u korištenju prosječnoga mjesečnog solarnog potencijala u proračunima, zbog čega se utjecaj sporadičnih visokoenergetskih događaja može previdjeti. Budući da se zrakoplovne tvrtke diljem svijeta gotovo isključivo koriste softverom za procjenu doze koju prima njihova posada (zbog troškova i jednostavnosti), preporučljivo je u slučaju promjene u Sunčevoj aktivnosti retrospektivno preračunati dozu.

There are two main components of primary cosmic radiation relevant in terms of exposure for the general population: galactic and solar. Both mostly involve high energy protons and depend on the Sun’s activity and the Earth’s magnetic field. Solar activity defines the energy delivered, whereas the Earth’s magnetic field deflects particles from entering the atmosphere. While the atmosphere protects life on Earth from primary cosmic radiation, it is also the source of secondary cosmic radiation, which mainly consists of photons, protons, neutrons, and charged and uncharged pions and muons. The neutron component dominates the hadron cascade at lower altitudes in the atmosphere (which coincides with the altitudes of commercial flights) as a result of their longer mean free path (1). Neutrons arise in nuclear reactions between high energy protons and atmosphere constituents. High energy protons, which induce neutron fields, are under the influence of the Earth’s magnetic field. As a result, neutron fields have a smaller fluence rate in the equatorial region than in the polar region. This is the reason why radiation doses change with a flight route.

The neutron component of the secondary cosmic radiation is also highly related to altitude. Neutrons contribute with around 50 % to the total dose equivalent rate, but this percentage varies between 40 and 80 % with altitude, latitude, and phase in the solar cycle (2). Secondary cosmic radiation on the Earth’s surface contributes with about 10 % of the total effective dose from natural sources (where ^222^Rn accounts for over 50 %). At higher altitudes the secondary cosmic radiation field becomes dominant. Typically, commercial aircraft altitudes are between 6,100 and 12,200 m, and the dose rate doubles with every 1800 m increase in altitude. The dose received on a particular flight depends on the altitude, latitude, and time of flight. The typical total dose rate for flights at 50 ° latitudes (intercontinental flights between Europe and North America or Asia) at standard flight altitudes is in the range of 4–8 μSv/h, but in some cases it can reach 10 μSv/h. The average total dose rate for flights at lower latitudes and for short-haul flights is around 3 μSv/h. Flights at high latitudes result in higher dose than flights in the equatorial region due to the geomagnetic effect (expressed in the terms of magnetic rigidity) (3, 4). Therefore, the radiation field at aircraft altitudes can be expressed as a function of three variables: flight altitude, solar modulation strength, and magnetic rigidity cut-off (5, 6).

Our previous study (3) presented the measurements of neutron dose equivalent on board several international flights during the first half of the solar cycle 24, while the aim of this study was to measure neutron dose equivalent on several international flights in its second half (from maximum to minimum) and to clearly discern the influence of solar activity, flight altitude, and flight route.

## Materials and methods

For this study we used passive neutron dosimeters consisting of solid-state nuclear track etched detectors LR 115 or CR-39 (Dosirad/Algade, Bessines-sur-Gartempe, France and Intercast Europe Co., Parma, Italy, respectively) and 10B foil as a converter (7, 8, 9). Nuclear reaction between the neutron and the boron converter ^10^B (n, α) ^7^Li produces two charged particles: Li ion and an alpha particle that leave different tracks in the detector body. Li ions have a much shorter range than alpha particles and are easily discriminated during etching. The dosimeters were calibrated in the CERN-EU high-energy reference field (CERF) in terms of known neutron ambient dose equivalent H*(10) = 1.0 mSv (10). The calibration parameters enabled us to calculate the sensitivity coefficient and the uncertainty of the dosimeter, as follows (1):

$$k=\frac{H^{*}(10)}{D}=\frac{1.0mSv}{3285\frac{\text{ tracks }}{{cm}^{2}}}\;\;(0.30\pm 0.01)\;\;\frac{\mu Sv\;{cm}^{2}}{\text{ tracks }}$$

where *k* is the sensitivity coefficient and *D* density of the tracks left by alpha particles produced by nuclear reaction between neutrons and boron. Sensitivity coefficient *k* of the dosimeter enables us to calculate neutron ambient dose equivalent and assess average neutron dose rate regardless of detector exposure.

However, considering that the CERF field has a relatively higher thermal neutron component than the atmosphere at aircraft altitudes, Equation 1 may result in an overestimation of *k*. Another limitation of the dosimeter are large uncertainties in high energy neutron response (11), which may result in underestimation of neutron dose equivalent. Uncertainty of measurements and detector manipulation were determined as described in our earlier study (3).

We then compared our measurements with simulations obtained with the European Program Package for the Calculation of Aviation Route Doses (EPCARD version 5.4.3, Helmholtz Zentrum Munich, German Research Centre for Environmental Health, Neuherberg, Germany) (12, 13), a German Aviation Authority-approved software based on the FLUKA (from German *FLUktuierende KAskade*) general-purpose tool for calculating particle transport and interactions with matter (14, 15). The EPCARD program simulates a flight (with time resolution of 1 min) in the “quasi-real” radiation field of cosmic secondary particles. It uses a large-scale set of “fluence-to-dose” conversion coefficients to calculate dose quantities (in μSv) in terms of ambient dose equivalent [H^*^(10)] and effective dose.

**Figure 1:**
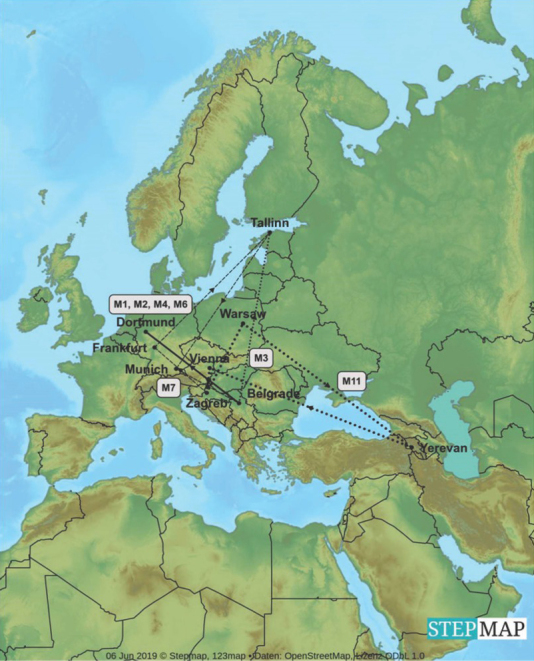
European flights on which dosimeter measurements were taken [map created with the StepMap software (16)]

**Figure 2:**
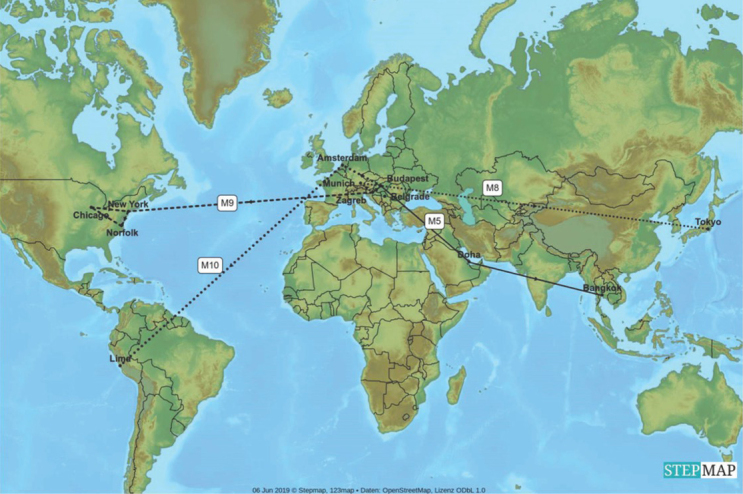
Intercontinental flights on which dosimeter measurements were taken [map created with the StepMap software (16)]

To determine neutron ambient dose equivalent as a measure of aircraft crew and passenger exposure passive dosimeters were taken on board of eleven international flights – shorter within Europe (M1, M2, M3, M4, M6, M7, M11, Figure 1) and longer between continents (M5, M8, M9, M10, Figure 2). As a standard procedure, the measurements do not include 15 min for the take-off and 15 min for landing.

## Results and discussion

Table 1 shows the results of passive dosimeter measurements and calculated average neutron ambient dose rates. The dose rate was calculated to easily compare measured and simulated values with known effects on the dose rate, such as the geomagnetic effect. Higher flight altitudes correlated with intercontinental flights and lower altitudes with European flights.

**Table 1:** Overview of the data obtained by measurements and simulations for the eleven flights studied. All of the measured results are written with associated uncertainties

Measurement code/flight route	Date of flight	Average neutron track density (track/cm^2^)	Measured neutron ambient dose (μSv)	Calculated neutron ambient dose rate (μSv/h)	ambient Total dose equivalent obtained by using EPCARD (μSv)	Total ambient dose rate obtained by using EPCARD (μSv/h)
M1: Belgrade–Dortmund–Belgrade	November 2014	11.9	3.6±1.4	0.90±0.83	20	5
M2: Belgrade–Dortmund–Belgrade	December 2014/January2015	42.0	13±6.8	2.9±1.5	20	5
M3: Belgrade–Tallinn–Belgrade	March 2015	81.6	25±15	4.1±3.7	32	5.3
M4: Belgrade–Dortmund–Belgrade	March 2015	11.2	3.4±1.4	0.85±0.92	20	5
M5: Zagreb–Budapest–Bangkok–Doha–Doha–Budapest–Zagreb	May 2015	82.6	25±6.5	1.0±0.3	62	2.4
M6: Belgrade–Dortmund–Belgrade	June 2015	15.6	4.7±2.4	1.2±0.9	20	5
M7: Zagreb–Frankfurt–Tallinn–Munich–Belgrade	May 2016	117.1	35±8.1	4.1±1.4	17	2
M8: Zagreb–Munich–Tokyo–Munich–Zagreb	December 2016	360.0	108±19	4.3±1.2	154	6.2
M9: Belgrade–New York–Norfolk–Chicago–New York–Belgrade	July 2017	188.9	57±6.4	2.4±0.4	166	6.9
M10: Budapest–Amsterdam–Lima–Amsterdam–Budapest	October 2017	60.5	18±6.4	0.65±0.20	108	3.9
M11: Zagreb–Warsaw–Yerevan–Vienna–Zagreb	October 2017	23.5	7.1±5.9	0.71±0.63	37	3.7

**Figure 3:**
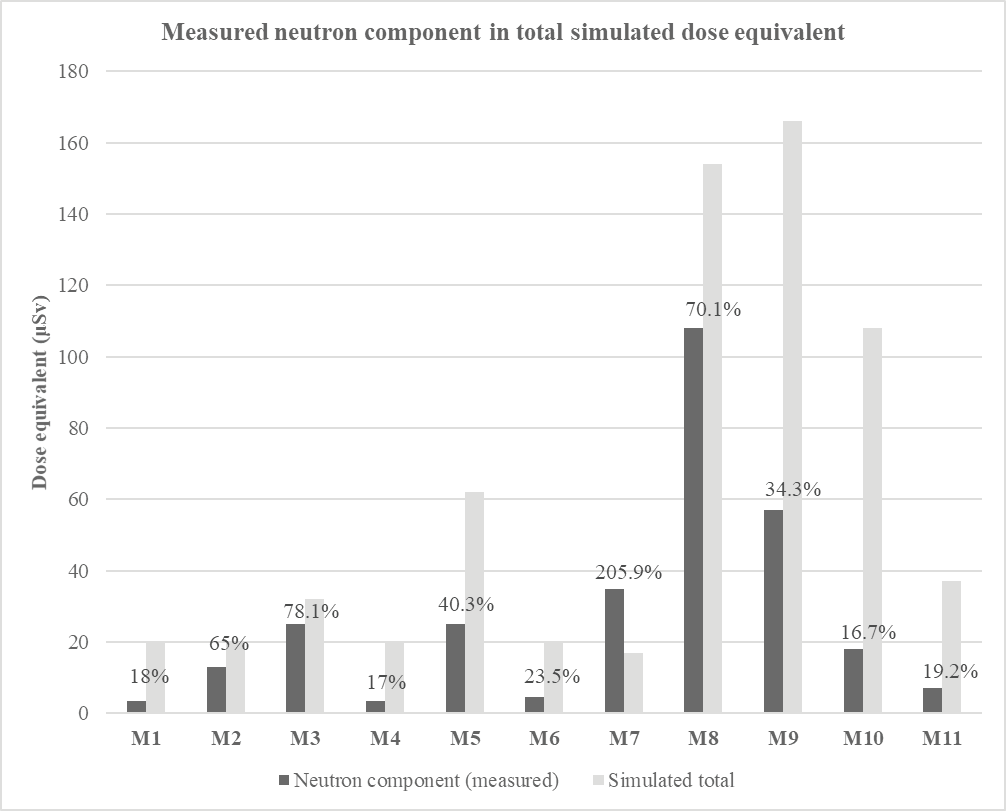
Measured neutron ambient dose equivalent (dark grey colour) and its percentage in the total EPCARD-simulated ambient dose equivalent (light grey colour; software). M1 to M11 designations correspond to flights in Table 1

Figure 3 shows the proportion of neutron ambient dose equivalent (obtained by measurement) in the total ambient dose equivalent (obtained by EPCARD software simulation). It also clearly shows that our measurements are outside of the widely accepted neutron contribution range of 40–80 %. We assumed that these discrepancies were the result of solar influence and checked them against specific flight times. We also checked the data from the Oulu neutron monitor (17, 18). Both confirmed our assumption. All of the flights were taken in the second part of the Solar cycle 24, that is, between 2014 (solar maximum) and 2018 (solar minimum). During the solar maximum, Sun releases sudden outbursts of energy and matter, which are called flares or solar proton events. These events can last for several hours to several days, and their energy can vary over several orders of magnitude. Energies that are high enough to drive particles deeply into the Earth’s atmosphere and increase radiation at aircraft altitudes can be observed with neutron monitors from the ground and are called ground level enhancements (GLE). During the strongest events, neutron flux can be several dozens of times higher than usual. In addition, when solar activity is weak, the flux of the galactic component of the primary cosmic radiation is expected to increase, which, in turn, increases radiation in the atmosphere. It is expected that the annual radiation dose during the solar minimum of the solar cycle 24/25 (five years) will be 19 % higher than in the last cycle (19).

However, sometimes solar flares with increased flux of charged particles flowing towards the Earth deflect protons of the galactic component of the primary cosmic radiation and therefore decrease neutron flux of the secondary cosmic radiation in the atmosphere. Such events are called Forbush decreases and may lower neutron flux by more than 20 % (20, 21). A case in point are our M1, M2, M4, and M6 measurements taken on the Belgrade–Dortmund flights at different times and therefore solar activities. The M2 measurement had much higher proportion of neutron dose rate than the other three measurements, as it occurred at very high solar activity (with 150 sunspots and several large solar proton events). This increase in neutron flux was also confirmed by the Oulu neutron monitor. The remaining three measurements (M1, M4, and M6) coincided with the opposite effect on the neutron flux (Forbush decrease).

Another measurement that stands out is M7 (Zagreb–Frankfurt–Tallinn–Munich–Belgrade), with an extreme jump above the simulated total ambient dose equivalent. It coincided with an intense G2-class solar flare reported by the NASA’s Solar Dynamics Observatory on 13 May 2015 that caused geomagnetic storms around the poles (22). The EPCARD software simulation, however, did not include this peak, as it averages solar potential over a month. The geomagnetic effect on the dose rate can also be spotted if we compare the flights at equatorial routes (M5 and M10) and the flights at polar routes (M8 and M9).

## Conclusion

Our findings confirm the strong influence of solar activity on both the increase or decrease of the neutron component of ambient dose equivalent we already reported earlier (3). The EPCARD software (and other similar programs) used by airlines to monitor radiation exposure run the risk of undermining actual doses by averaging solar potential over a month. A question remains whether flight crews are exposed to higher doses of radiation over the periods of high solar activity or unusual solar behaviour than simulations show. To address this main drawback and a source of possible underestimation of the dose received by the crew, dose estimations should therefore take into account flare (SPE) recordings by neutron monitors retrospectively.

## References

[1] Bartlett D (2004). Radiation protection aspects of the cosmic radiation exposure of aircraft crew. Radiat Prot Dosim.

[2] UNSCEAR 2008 Report. Sources and effects of ionizing radiation; United Nations Scientific Committee on the Effects of Atomic radiation. Vol I - Report to the General Assembly with Scientific Annexes A and B.

[3] Poje M, Vuković B, Radolić V, Miklavčić I, Planinić J (2015). Neutron radiation measurements on several international flights. Health Phys.

[4] Vuković B, Radolić V, Lisjak I, Vekić B, Poje M, Planinić J (2008). Some cosmic radiation dose measurements aboard flights connecting Zagreb Airport. Appl Radiat Isot.

[5] Poje M, Vuković B, Radolić V, Miklavčić I, Faj D, Varga Pajtler M, Planinić J (2012). Mapping of cosmic radiation dose in Croatia. J Environ Radioact.

[6] Paschoa AS, Steinhäusler F (2010). Technologically Enhanced Natural Radiation. Radioactivity in the Enviroment.

[7] Horwacik T, Bilski P, Olko P, Spurny F, Turek K (2004). Investigations of doses on board commercial passenger aircraft using CR-39 and thermoluminiscent detectors. Radiat Prot Dosim.

[8] Bartlett DT, Tanner RJ, Hager LG (2002). The high energy neutron response characteristics of a passive survey instrument for the determination of cosmic radiation fields in aircraft. Radiat Prot Dosim.

[9] Vuković B, Radolić V, Miklavčić I, Poje M, Varga M, Planinić J (2007). Cosmic radiation dose in aircraft - a neutron track etch detector. J Environ Radioact.

[10] Mitaroff A, Silari M (2002). The CERN-EU high-energy reference field (CERF) facility for dosimetry at commercial flight altitudes and in space. Radiat Prot Dosim.

[11] Goldhagen P, Clem JM, Wilson JW (2004). The energy spectrum of cosmic - ray induced neutrons measured on an airplane over a wide range of altitude and latitude. Radiat Prot Dosim.

[12] Mares V, Maczka T, Leuthold G, Rühm W (2009). Air crew dosimetry with a new version of EPCARD. Radiat Prot Dosim.

[13] (2010). EPCARD 5.4.3. European Program Package for the Calculation of Aviation Route Doses.

[14] Bohlen TT, Cerutti F, Chin MPW, Fasso A, Ferrari A, Ortega PG, Mairani A, Sala PR, Smirnov PR, Vlachoudis V (2014). The FLUKA Code: developments and challenges for high energy and medical applications. Nucl Data Sheets.

[15] Ferrari A, Sala PR, Fasso A, Ranft J (2005). FLUKA: a multi-particle transport code, CERN-2005-10, INFN/TC_05/11, SLAC-R-773.

[16] StepMap Software for creating maps.

[17] Spaceweather Forecast of solar flares and geomagnetic storms, plus daily status of the Sun.

[18] Oulu Neutron Monitor.

[19] Miyake S, Kataoka R, Sato T (2017). Cosmic ray modulation and radiation dose of aircrews during the solar cycle 24/25. Space Weather.

[20] Heinrich W, Roesler S, Schraube H (1999). Physics of cosmic radiation fields. Radiat Protect Dosim.

[21] Hubert G, Pazianotto MT, Federico CA, Ricaud P (2019). Analysis of the Forbush decreases and ground-level enhancement on September 2017 using neutron spectrometers operated in antarctic and midlatitude stations. JGR: Space Physics.

[22] Spaceweather Geomagnetic storms.

